# The effect of COVID‐19 vaccination on multiple sclerosis activity as reflected by MRI

**DOI:** 10.1002/brb3.3587

**Published:** 2024-06-28

**Authors:** Esther Ganelin‐Cohen, Chen Buxbaum, Noam Bosak, Shani Sobol, Adi Vaknin‐Dembinsky, Mark A Hellmann, Adi Wilf‐Yarkoni, Keren Regev, Elizaveta Pustovoyt, Alla Shifrin, Yair Wexler, Ayal Rozenberg

**Affiliations:** ^1^ Neuroimmunological Clinic Institute of Pediatric Neurology, Schneider Children's Medical Center of Israel Petah Tikva Israel; ^2^ Sackler School of Medicine Tel Aviv University Tel Aviv Israel; ^3^ Department of Neurology Rambam Health Care Campus Haifa Israel; ^4^ Unit for Neuro‐Immunology, Multiple Sclerosis & Cell Therapy, Department of Neurology Hadassah Medical Center Jerusalem Israel; ^5^ Department of Neurology, Rabin Medical Center Beilinson Hospital Petah Tikva Israel; ^6^ The Neuroimmunology and Multiple Sclerosis Unit, Neurology Institute Tel Aviv Sourasky Medical Center Tel Aviv Israel; ^7^ School of Neurobiology, Biochemistry and Biophysics, The George S. Wise Faculty of Life Sciences Tel‐Aviv University Tel Aviv Israel; ^8^ Neuroimmunology Laboratory, Department of Neurology, Rambam Health Care Campus and Ruth and Bruce Rapaport Faculty of Medicine, Technion Israel Institute of Technology Haifa Israel

**Keywords:** COVID‐19, disease‐modifying therapy, expanded disability status scale, mRNA vaccine, relapsing‐remitting multiple sclerosis

## Abstract

**Introduction:**

Examining the safety of theBNT162b2 mRNA vaccine in multiple sclerosis (MS) patients remains inconclusive, particularly regarding the potential for disease exacerbations. This study aims to assess the effects of BNT162b2 COVID‐19 vaccination on disease activity in MS patients through sequential MRI imaging.

**Methods:**

A retrospective study of 84 MS patients from five Israeli hospitals was conducted. MS lesion load was determined from three brain MRI scans, one postvaccination and two prevaccination scans. A post hoc analysis compared subgroups featuring vaccinated and unvaccinated patients respectively, with early onset MS.

**Results:**

The cohort included 70 women with early onset (mean age 16.4 ± 0.8 years) and adult onset (mean age 34.9 ± 1.1 years) MS. Among the early onset group, vaccinated patients showed an increased risk of new lesions (*p* = .00026), while there was no increased risk among adult‐onset patients. Additionally, a comparison between early onset vaccinated and nonvaccinated groups revealed a higher risk of increased lesions in the vaccinated group (*p* = .024).

**Discussion:**

Overall, the study suggests that the BNT162b2 vaccine is generally safe in MS patients, with no association found between vaccination and new lesions in most patients. However, close MRI follow‐up is recommended for early‐onset MS cases to monitor lesion development.

## Graphical abstract

Our findings support the safety of BNT162b2 COVID‐19 vaccination is safe for most MS patients, except for young patients who demonstrate increased lesions after vaccination. However, further studies with a larger sample size of patients who were not vaccinated or recovered from COVID‐19 are needed. An extensive consideration of risk factors is needed to clarify the relationship between immunization and MS activity.

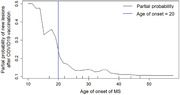

## 1 INTRODUCTION

The COVID‐19 pandemic presented new challenges across healthcare, specifically shifting the management of autoimmune diseases amongst many other diseases. The virus triggers an autoimmune reaction known as a cytokine storm, the leading pathogenic cause for numerous pathologies (Ragab et al., 2020).

Accounting for the complex features of the virus, the SARS‐CoV‐2 spike protein is generally identified as the most significant antigen target as previous studies demonstrate the efficacy of antibodies to spike proteins in SARS‐CoV and MERS‐CoV (Alharbi et al., 2017; Coleman et al., 2017; He et al., 2004). Of note, concerns have been raised regarding the potential for dysfunctional immune responses, allowing for autoimmune disease onset and exacerbation. In the schema of molecular immunology, similarities in the structures of SARS‐CoV‐2 spike protein and mammalian peptides have been outlined (Kanduc & Shoenfeld, 2020), and varying degrees of reactivity between the SARS‐CoV‐2 spike protein antibody and tissue proteins have been demonstrated in vitro (Vojdani & Kharrazian, 2020). Additionally, it has been postulated that the binding of pattern recognition receptors (PRRs) to the viral mRNA before translation may lead to up‐regulation of proinflammatory cascades, a vital measure of autoimmune flare‐ups (Talotta, 2021).

Regarding COVID‐19 vaccination, studies on patient populations with existing autoimmune conditions have not found an increase in exacerbations following vaccination with mRNA vaccines (Braun‐Moscovici et al., 2021; Connolly et al., 2021; Furer et al., 2021). A case series from Israel, the United Kingdom, and the United States reported on disease flares and new‐onset rheumatic conditions following vaccination (Watad et al., 2021). However, the lack of data on the rate of incidence makes it difficult to assess the probability of a causal link.

Known as one of the more common immune‐mediated diseases, multiple sclerosis (MS) is an autoimmune inflammatory disease with multifactorial etiologies (Remez et al., 2021). The role of vaccination is of interest as it is one of the extensively studied risk factors for disease exacerbation in MS (Loebermann et al., 2012; Mailand & Frederiksen, 2017). While initial reports on the efficacy COVID‐19 mRNA vaccines in patients with MS have been published, the existing literature has been notably limited in addressing vaccines safety concerns (Achiron et al., 2021; Coyle et al., 2021; Guerrieri et al., 2021). Several studies, including a case series, underscored clinical and radiological relapses in thirteen MS post COVID‐19 vaccination, particularly with the BNT162b2 mRNA vaccine, raising apprehensions about vaccine safety implications (Nistri et al., 2021). Additionally, association between COVID‐19 vaccination and inflammatory CNS diseases, including initial manifestations of MS after Pfizer's COVID‐19 vaccine (Havla et al., 2021; Nistri et al., 2021) and MS relapse following different vaccines for COVID‐19, has been documented (Etemadifar et al., 2021; Khayat‐Khoei et al., 2021).

However, a recent meta‐analysis, encompassing 19 observational studies shed light on a more reassuring perspective (Stefanou et al., 2023). The analysis reveals a pooled proportion of 1.9% (95% CI 1.3%−2.6%) of MS patients experiencing relapse within a mean time interval of 20 days from vaccination. Importantly, this relapse risk appears independent of type of SARS‐CoV02 vaccine administered. The study further notes that transient neurological worsening occurred in 4.8% (95% CI 2.3%−8.1%) of patients’ postvaccination.

Contrary to earlier concerns, this meta‐analysis suggests that COVID‐19 vaccination does not seem to increase the risk of relapse or serious adverse events in MS patients. Notably, the study primarily focused on adults, as indicated by the mean age of 43.3 years (95% CI 40–46.6), and specifically investigated clinical relapses without focus on imaging.

While the meta‐analysis provides valuable insights, it is crucial to recognize that it may not comprehensively capture potential nuances in specific subgroups, such as individuals with new‐onset and younger cases of MS. Further investigation within these specific demographic subgroups is essential to deepen our understanding of the implications of COVID‐19 vaccination, particularly concerning factors beyond clinical relapses, such as the potential for Magnetic Resonance Imaging (MRI) changes.

For the last two decades, MRI scans have been an integral criterion in the diagnosis of MS (Thompson et al., 2018). MRI is a major component of the routine medical management of patient with MS, playing a pivotal role in the assessment of disease activity (Eran et al,. 2018), response to treatment, and monitoring disease activity, even in the absence of clear clinical manifestations (Lövblad et al., 2010).

In this study, we aimed to investigate whether COVID‐19 vaccination influences the individual disease course of MS patients. To address this question, we chose to focus on MRI dynamics, regardless of clinical exacerbations, as a sensitive indicator of disease activity.

## 2 MATERIALS AND METHODS

### 2.1 Source of data

This is a retrospective, uncontrolled study, based on medical records from five medical centers, including documented MRI reports of each scan conducted by neuro‐radiologists of each respective center.

### 2.2 Study population

Five neuro‐immunological centers in Israel participated in this study: Rambam Health Care Campus, Rabin Medical Center, Schneider Children's Medical Center, Tel Aviv Sourasky Medical Center, and Hadassah Medical Center. The study was approved by each institution's respective review boards (260‐21‐RBM, 0382‐21‐RMC, 0277‐21‐RMC, 0597‐17‐TLV, 0332‐21‐HMO).

In each center, the neuro‐immunology specialists screened their MS patients’ records for the dates of their BNT162b2 mRNA COVID‐19 vaccination. The diagnosis of MS was determined according to the 2010 and 2017 revised McDonald criteria and MAGNIMS consensus guidelines (Filippi et al., 2016; Thompson et al., 2018). Patients eligible for inclusion were consecutively selected from our clinical database during routine follow‐up, with exclusion criteria applied to ensure adherence to specified requirements. Inclusion criteria were:

#### 2.2.1 Study group (vaccinated MS patients)

1. Completed two doses of BNT162b2 mRNA COVID ‐19 vaccinations since 2020.
2. Underwent routine brain MRI scans between 2019 and 2021, encompassing two scans during the year preceding the first dose (“pre‐vaccine MRI”). A third MRI within 3 months after the second vaccination (“post vaccine MRI”). Two‐time intervals between the three scans approximately equal. Recorded neuro‐radiologist's interpretation for each patient's MRI series.

#### 2.2.2 Control group (early onset MS patients under 20 (not vaccinated)

(1) Diagnosed with MS before the age 20.
(2) Did not receive vaccination.
(3) Underwent two sequential MRIs in the same period as those under 20 in the vaccinated group.

Patients with a positive documented COVID‐19 test were excluded, as well as those with any documented evidence of other CNS involvement, such as seizures or encephalitis. Additionally, individuals who received another specific vaccination were excluded from the study.

As Relapsing‐remitting MS (RRMS) patients constitute most MS patients, the final statistical analyses only included RRMS patients.

### 2.3 Study outcomes

As we aimed to use brain MRI dynamics to determine whether the vaccinations affected individual disease course, parameters indicating MS activity were pertinent to investigation.

We chose not to include spinal MRI in our analysis, aligning with common recommendations for routine follow‐up, where annual brain MRI scans are standard. Although spinal MRI is recommended in diagnosis and on clinical demand, it is not part of the default routine, reflecting standard practice (Wattjes et al., 2021).

The primary outcome measure during the postvaccine period was the observed deteriorating change in the third MRI, as compared to the prevaccine MRI.

In the context of early‐onset MS, secondary outcomes exclusively assessed the count of new lesions or newly enhancing lesions observed between consecutive MRIs in both the nonvaccinated control group and the early‐onset MS study group.

These parameters were retrospectively collected from the documented reports of the scans. Routine answers provided by neuro‐radiologists blinded to the patient's vaccination status bypass potential bias MRI interpretation influenced by considerations for COVID‐19 vaccines.

For patients meeting the inclusion criteria, each center provided the following parameters: clinical diagnosis, age, age at onset, gender, disease duration, Expanded Disability Status Scale (EDSS) score, disease duration, type of disease‐modifying therapies (DMT) in use, and presence of an oligoclonal band (if examined).

### 2.4 Statistical methods

For comparison between the various groups of MS patients, the measured outcome was the presence of new lesions in their last MRI scan. Significance was determined by setting the probability threshold at .05 and was assessed using Fisher's Exact Test. Nonparametric 95% confidence intervals were calculated for the reported odds ratios (Fay, 2010). A likelihood‐based 95% confidence interval was calculated for the probability of new lesions within each group.

### 2.5 Variable selection and age of onset cutoff

Age of onset of MS was determined as the most important variable to differentiate between vaccinated MS patients who developed new lesions and those who didn't develop. Variable importance was assessed using the random forest algorithm (combines the output of multiple decision trees to reach a single result) (Breiman, 2001), performed using the “*randomForest*” R package (RColorBrewer & Liaw, 2018) with parameters *m_try _* = 2 and 500 trees. Other parameters yielded equivalent results. Partial dependence of the existence of new lesions on the age of onset rapidly decreased for ages up to 20 years old. Therefore, the effect of the vaccine was likely to be the most severe below at ages below 20 years old. Comparison between vaccinated patients and unvaccinated control patients was thus limited to an age of onset of up to 20 years of age.

## 3 RESULTS

### 3.1 RMSS research population

Of the 84 RRMS patients in the statistical analysis, 70 were women. The mean age of the adult‐onset group was 34.9 ± 1.1 years and an average of 9.5 ± 1 years of disease, the mean age of the early‐onset group was 16.4 ± 0.8 years old at onset of disease, and the average duration of disease of 5.6 ± 1.5 years. Average EDSS was 1.88 ± 0.42 for the early‐onset group and 1.98 ± 0.21 for the adult group respectively. Data are presented in Table 1.

**TABLE 1:** Characteristics of the study sample of 84 MS patients who were vaccinated against COVID‐19.

All vaccinated patients (early onset cutoff = 20 years)		Age of onset of MS (years)		Total
		early onset 9–20	Adults onset 21+	
Variable		14	70	84
Age (years)	Mean ± SEM (Q1, Median, Q3)	16.4 ± 0.8 (15, 16.0, 18.8)	34.9 ± 1.1 (27.3, 32, 43.8)	31.8 ± 1 (24, 30, 39)
Duration of MS (years)	Mean ± SEM (Q1, Median, Q3)	5.6 ± 1.5 (1, 4, 8.5)	9.5 ± 1.0 (3, 6, 14)	8.9 ± 0.8 (3, 6, 13)
Gender	Female	13 (19%)	57 (81%)	70
	Male	1 (7%)	13 (93%)	14
Presence of OCB	Yes	13 (25%)	38 (75%)	51
	No	0 (0%)	9 (100%)	9
	Missing	1	23	24
EDSS	Mean ± SEM (Q1, Median, Q3)	1.88 ± 0.42 (1, 1, 2)	1.98 ± 0.21 (1, 1.5, 3)	1.96 ± 0.19 (1, 1.5, 3)

*Note*: Distribution of MS treatment is presented in Table S1.

When examining the presence of oligoclonal bands, the early‐onset age group had all 13 participants classified as OCB positive. In contrast, 38 out of 47 were positive in the adult‐onset group. However due to the small sample size, we couldn't determine statistical significance.

### 3.2 Vaccination effect on MRI dynamics—comparison according to the age of onset MS

**FIGURE 1:**
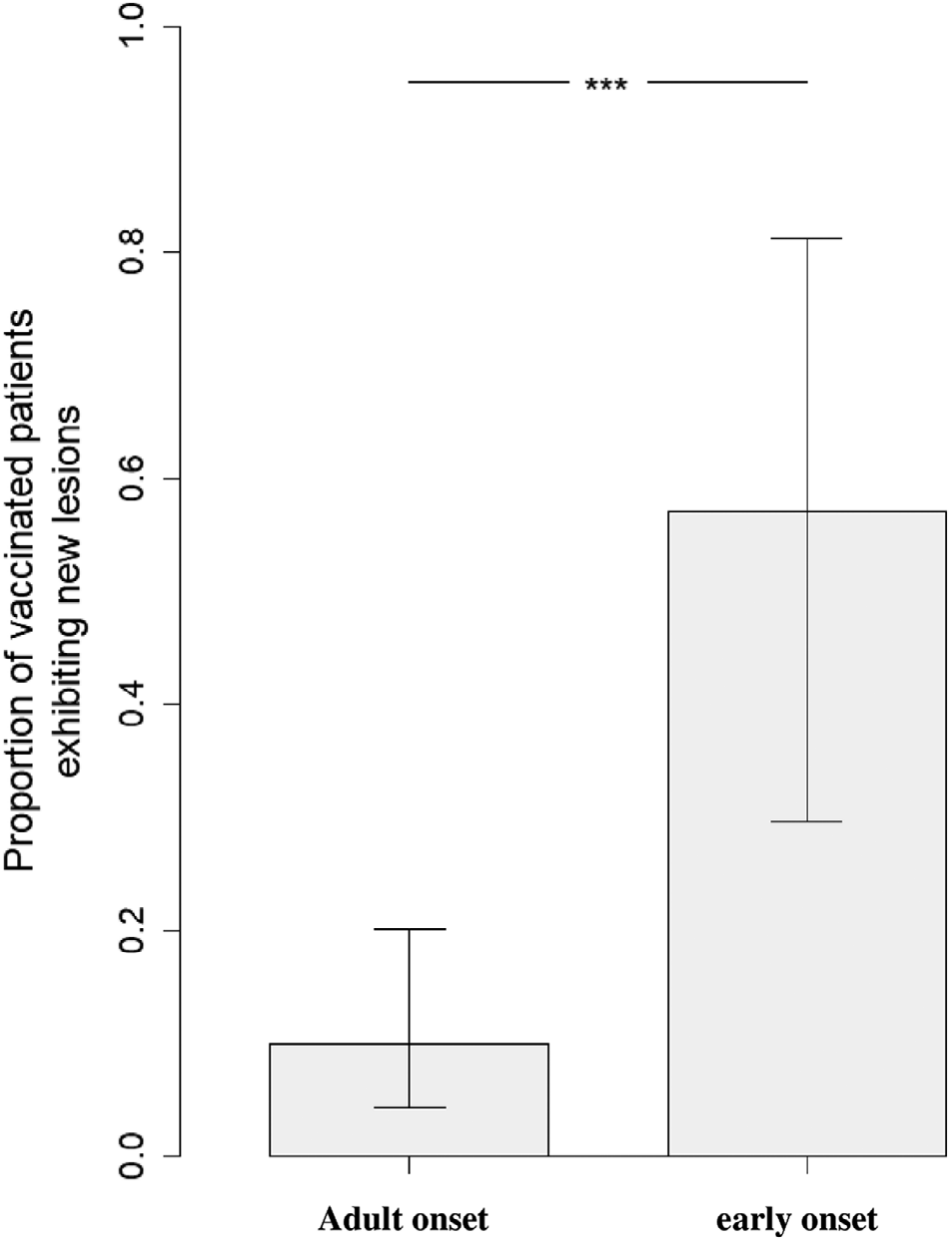
Patients were divided into two groups—early‐onset (age of onset at most 20, *n* = 14) and adults (age of onset 21+, *n* = 70). Increased lesions in vaccinated patients among early onset patients. OR = 12.00 (95% CI 2.99–45.35). RR = 5.71 (95% CI 2.58–12.68). The difference was statistically significant at a .05 level with *p* = .00026 (“***” in the plot below). Statistical difference was assessed using Fisher's exact test, and 95% confidence intervals were likelihood‐based.

According to the age of onset MS, there is a substantial difference in response to COVID vaccination. For example, Figure 1 indicates a significant increase in the chance of developing a new lesion after COVID vaccination in the early age of onset group in comparison to the adult onset (*p* = .00026*).

**FIGURE 2:**
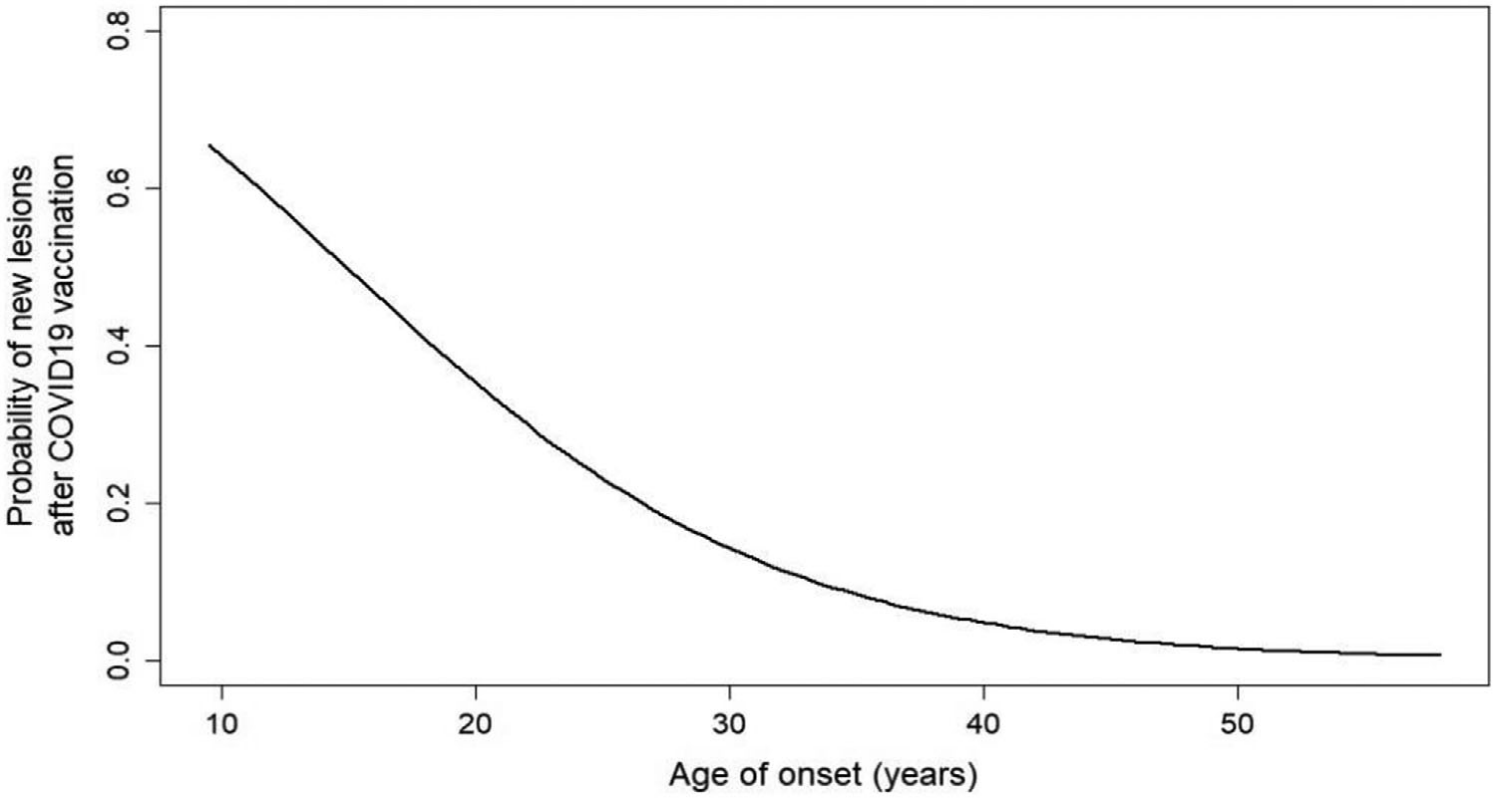
The following plot shows the probability of finding new lesions for every age of onset of MS present in the study. The probabilities were estimated using a logistics regression model (black line) with age of onset as the only covariate. The age of onset effect was significant (*p* = .00021***, log‐likelihood ratio test). The estimated odds ratio for an “older” patient compared to a “younger” patient, whose difference in age of onset is 10 years, is 0.30.

The following plot shows the probability of finding new lesions for every age of onset of MS present in the study. The probabilities were estimated using a logistics regression model (black line) with age of onset as the only covariate. The age of onset effect was significant (*p* = .0002***), based on a log‐likelihood ratio test (Figure 2).

### 3.3 Vaccination effect on MRI dynamics according to different parameters

We compared different parameters such as the presence of oligoclonal bands, gender and DMT type to determine if they influence the chance of developing new lesions. The positive oligoclonal test did not correlate significantly with the increased lesions detected following immunization. Nevertheless, it is worth mentioning that within the early onset group, the patients that had dynamic changes on MRI all tested positive for the presence of oligoclonal bands.

Gender did not pose as a significant risk factor for lesion load postvaccination. The different DMT models are important variables, but we could not calculate a statistical significance due to the small size of the cohorts.

### 3.4 Vaccinated and unvaccinated groups

By means of the post hoc analysis for the early onset RRMS group, we compared the difference between the vaccinated and unvaccinated groups, matched by demographics and their performance of two MRI scans at a parallel time and equal intervals of 6 months between two MRI scans.

**FIGURE 3:**
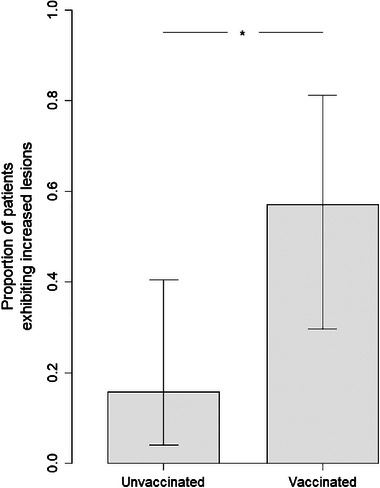
The probability for new lesions was compared between the group of vaccinated early‐onset patients (*n* = 14) and a control group of unvaccinated early‐onset patients (*n* = 19). Increased lesions in pediatric vaccinated patients. OR = 7.11 (95% CI 1.29–49.16). RR = 3.62 (95% CI 1.28–10.20). *p* Value of fisher's exact test was .024. The difference was statistically significant at a 0.05 level with *p* = .024 (“*” in the plot below).

When comparing the vaccinated versus nonvaccinated groups, there is a significant increase in the number of patients with increased lesions among the vaccinated group (*p* = .0246) (Figure 3).

The demographical and clinical data of the early onset study cohort for vaccinated and unvaccinated groups are presented in Tables S2 and S3.

### 3.5 Formula computing the chance of new lesions according to three parameters model

**FIGURE 4:**
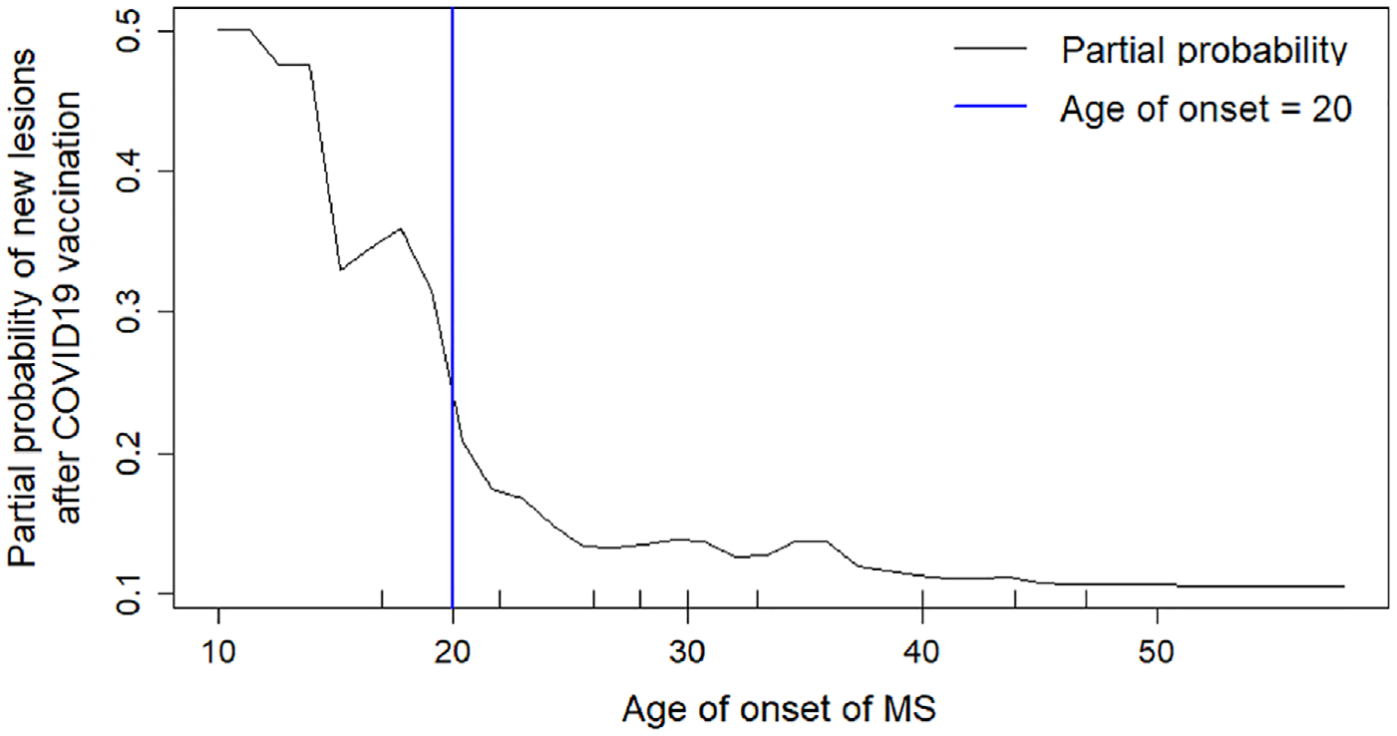
Partial dependence plot for the age of onset variable following the random forest algorithm. 20 years was taken as cutoff to differentiate “early onset” and “adult onset” patients.

The following “Partial Dependence plot,” based on the RF model, estimates the probability of a positive outcome (new lesions) for each value of the age of onset. The probability of new lesions post vaccination at a young age of onset is much higher than at an older age (Figure 4).

## 4 DISCUSSION

The association between vaccination and MS exacerbation is not well established, raising concern for vaccination safety in MS patients. Considering the accumulating data of post‐COVID‐19 vaccination side effects involving the central nervous system (CNS) in the general population, the concern is amplified in consideration of MS patients. Israel was among the first countries to employ a vaccination drive, with a large percentage of the population receiving a relatively early coverage of two vaccinations. The well‐documented vaccination drive enables a systematic examination of the vaccinated MS patient population, as compared to cases that appear sporadically.

Our results show that two doses of the BNT162b2 mRNA vaccine significantly impacted the early‐onset disease group in comparison to the adult‐onset group. In other parameters such as the presence of oligoclonal bands and gender, there was no impact on the chance of developing new lesions. Additionally, DMT displayed a notable effect in the partial dependence plot; however, statistical significance could not be calculated due to the cohort's subdivision into many small groups.

In contrast, our primary study population of adult‐onset had stable disease progression correlated with extensive literature that ruled out most vaccinations as risk factors for MS relapses (Loebermann et al., 2012; Mailand & Frederiksen, 2017), with COVID‐19 vaccinations as part of the rule rather than the exception (Iacobaeus et al., 2020). Thus, our study provides further evidence to prove the COVID‐19 vaccine's safety profile for the general MS population. However, specific populations, specifically young MS patients, are at increased risk of having subclinical changes postvaccination.

Our finding concerning the possible risk of disease activity after vaccination among early‐onset MS patients raises an intriguing thought in the pathophysiology of MS. It is well known that MS shows a more aggressive course, higher annual relapse rate, and more significant brain T2 lesion load when diagnosed at a younger age as compared to disease course that begins at an older age (Ghezzi et al., 2017).

Furthermore, patients with early‐onset MS are reported to have increased innate immune responses due to the high production of NK cells compared to older patients (Saccà et al., 2019). In children with MS, NK cells produce IL‐17A in the early stages of infection (Sormani et al., 2021), demonstrating a strong correlation between IL‐17A, a proinflammatory cytokine, and new lesions in MRI scans (Lotan et al., 2021). Another study revealed a 50% increase in acute axonal damage in early active demyelinating lesions in children compared to adults (Shaw et al., 2013). We assumed that those pathophysiological differences cause an increased sensitivity of early‐onset MS patients to external triggers such as vaccination. This theory must be better substantiated as the current literature requires more supporting publications on the pathophysiological connection between vaccination and autoimmune inflammation. A population‐based study may counter the supposed correlation as it showed no particular increase in risk from exposure to vaccination (Filippatos et al., 2021).

In a related study regarding MS patients and their response to influenza vaccination, one patient who experienced a relapse also showed dynamic changes in his imaging results a year prior (Hedegaard et al., 2008). This observation, based only on one patient, stands in contrast with our findings where the cohort of young patients had a stable disease course a year before vaccine administration, demonstrating that the progression is not part of their disease's natural history.

Potential limitations in our study include a small patient cohort and the singular focus on the vaccinated portion of the MS population, excluding consideration for those with a pediatric age of onset. Additionally, the study encompasses various DMTs used by the patients, but the limited number of distinct DMT groups poses a challenge in drawing nuanced conclusions. It is noteworthy that DMTs have diverse effects on individuals with MS due to differences in their mode of action. To address these constrains, a larger sample size is essential to match unvaccinated MS patients with controls, thereby improving statistical power and providing a more robust foundation for interpreting the study's finding.

The uncontrolled study methodology provides an opportunity to explore the disease course of MS at the individual level. Despite the limitations, our study successfully and independently demonstrates significant disease exacerbation after vaccination among early‐onset age on onset of disease, and no activity of disease among adult onset of disease. Two statistical methods support the pediatric‐onset results, while the adult's onset demonstrated by pre‐ and postmethodology showed only minimal activity postvaccination.

## 5 CONCLUSIONS

Our findings support the safety of BNT162b2 COVID‐19 vaccination is safe for most MS patients, except for young patients who demonstrate increased lesions after vaccination. However, further studies with a larger sample size of patients who were not vaccinated or alternatively recovered from COVID‐19. An extensive consideration of risk factors is needed to clarify the relationship between immunization and MS activity.

## AUTHOR CONTRIBUTIONS

**Esther Ganelin‐Cohen**: Conceptualization; data curation; investigation; methodology; project administration; supervision; writing—original draft. **Chen Buxbaum**: Data curation; formal analysis. **Noam Bosak**: Data curation; writing—original draft. **Shani Sobol**: Data curation. **Adi Vaknin‐Dembinsky**: Data curation; investigation. **Mark Hellmann**: Data curation. **Adi Wilf Yarkoni**: Data curation. **Keren Regev**: Data curation; investigation. **Elizaveta Pustovoy**: Data curation. **Alla Shifrin**: Data curation. **Yair Wexler**: Formal analysis; writing—review and editing. **Ayal Rozenberg**: Conceptualization; data curation; investigation; methodology; project administration; writing—original draft.

## FUNDING

There was no funding for this article.

## CONFLICT OF INTEREST STATEMENT

On behalf of all authors, the corresponding author states that there is no conflict of interest.

### PEER REVIEW

The peer review history for this article is available at https://publons.com/publon/10.1002/brb3.3587.

## Supporting information

Table S1: DMTs type distributions among the patients cohort study.

Table S2: Demographical and clinical data of the early onset MS vaccinated group.

Table S3: Demographical and clinical data of the early onset MS unvaccinated group.

## Data Availability

The data that support the findings of this study are available from the corresponding author upon reasonable request.
