# Supplementary material for: The effect of COVID‐19 vaccination on multiple sclerosis activity as reflected by MRI
Source: Brain Behav. 2024 Jun 28;14(7):e3587. doi: 10.1002/brb3.3587 (PMC11212002; doi:10.1002/brb3.3587)
Supplement: Supplementary file 1 — Table S1: DMTs type distributions among the patients cohort study. [file BRB3-14-e3587-s002.docx]

Appendix Table 1: DMTs Type Distributions Among the Patients Cohort

Study

| Type of treatment | Number of patients |
| --- | --- |
| Glatiramer Acetate | 5 |
| Interferon Beta | 7 |
| Dimethyl Fumarate | 19 |
| Diroximel Fumarate | 2 |
| Cladribine | 7 |
| Teriflunomide | 5 |
| Fingolimod | 5 |
| Ocrelizumab | 12 |
| Natalizumab | 7 |
| Alemtuzumab | 1 |
| Rituximab | 1 |
| no treatment | 13 |
