# Supplementary material for: The effect of COVID‐19 vaccination on multiple sclerosis activity as reflected by MRI
Source: Brain Behav. 2024 Jun 28;14(7):e3587. doi: 10.1002/brb3.3587 (PMC11212002; doi:10.1002/brb3.3587)
Supplement: Supplementary file 3 — Table S3: Demographical and clinical data of the early onset MS unvaccinated group. [file BRB3-14-e3587-s001.docx]

**Appendix Table 3**: **Demographical and Clinical Data of the Early onset MS**

**unvaccinated group.**

| Total Relapses | Last Relapse Before Vaccination (months) | Current DMT duration (months) | DMT Type | OCB | EDSS | Duration of MS Disease  (years) | Age of onset MS | Gender | Change in MRI | Serial Number |
| --- | --- | --- | --- | --- | --- | --- | --- | --- | --- | --- |
| 2 | 23 | 9 | 9 | n/a | 3 | 5 | 15 | 1 | 0 | 1 |
| 2 | 13 | 5 | 7 | n/a | 3.5 | 2 | 18 | 1 | 0 | 2 |
| 2 | 9 | 4 | 8 | n/a | 3 | 2 | 18 | 1 | 0 | 3 |
| 4 | 10 | 7 | 0 | 1 | 0 | 3 | 14 | 0 | 0 | 4 |
| 2 | 24 | 2 | 2 | 1 | 2 | 4 | 15 | 1 | 0 | 5 |
| 2 | 12 | 10 | 2 | 1 | 1 | 2 | 17 | 1 | 1 | 6 |
| 2 | 14 | 16 | 10 | 1 | 1 | 2 | 14 | 1 | 0 | 7 |
| 2 | 24 | 11 | 2 | 0 | 1 | 4 | 9.5 | 0 | 0 | 8 |
| 2 | 22 | 8 | 2 | 1 | 0 | 2.5 | 15.5 | 1 | 0 | 9 |
| 2 | 24 | 9 | 1 | 0 | 0 | 4.5 | 12 | 1 | 0 | 10 |
| 2 | 6 | 3 | 0 | 1 | 1 | 2 | 16 | 0 | 1 | 11 |
| 2 | 11 | 15 | 2 | 1 | 1 | 2.5 | 16.5 | 1 | 1 | 12 |
| 5 | 9 | 5 | 0 | 1 | 1 | 2 | 12.5 | 1 | 0 | 13 |
| 4 | 8 | 18 | 1 | 1 | 0 | 1.5 | 14.5 | 0 | 1 | 14 |
| 2 | 14 | 18 | 1 | 1 | 0 | 2 | 16 | 1 | 0 | 15 |
| 2 | 17 | 24 | 1 | 1 | 0 | 3 | 13.5 | 1 | 0 | 16 |
| 2 | 9 | 8 | 1 | 1 | 0 | 1.5 | 16.5 | 1 | 0 | 17 |
| 4 | 32 | 7 | 2 | 1 | 0 | 5.5 | 10.5 | 0 | 0 | 18 |
| 2 | 14 | 2 | 0 | 1 | 1 | 2 | 15.5 | 1 | 0 | 19 |

**G Gender**: man-0, female- 1.

**OCB**: negative-0, positive-1, n/a-not available.

**DMT Type**: Glatiramer Acetate -0, Interferon Beta -1, Dimethyl Fumarate -2, Diroximel Fumarate - 3,

Cladribine-4 Teriflunomide- 5, Fingolimod- 6, Ocrelizumab-7, Natalizumab- 8, Alemtuzumab- 9, other-10,

no treatment -11.

**Change in MRI**: 0- no change, 1- increased number of lesion or enhance lesion.
